# Social and physical environment effects on toileting disability among older adults in India

**DOI:** 10.1186/s12877-024-05198-5

**Published:** 2024-07-23

**Authors:** Joelle H. Fong, Y. Selvamani

**Affiliations:** 1 Lee Kuan Yew School of Public Policy, National University of Singapore, 469C Bukit Timah Road, Singapore, 259771 Singapore; 2 School of Public Health, SRM Institute of Science and Technology, Kattankulathur, Chennai, India

**Keywords:** Activity of daily living, Environment, Functional disability, Population aging, Toilets

## Abstract

**Background:**

To examine the prevalence of toileting disability among older adults in India and its association with broad aspects of the physical and social environment.

**Methods:**

We use data from the inaugural wave of the Longitudinal Ageing Study in India and focus on adults aged 65 and older (*N* = 20,789). We draw on the disablement process model and existing frameworks to identify environmental factors and other risk factors that may be associated with toileting disability. Hierarchical logistic regressions are implemented to analyze the health impacts from physical and social environment characteristics.

**Results:**

One in five older Indian adults had difficulties with toileting, and the prevalence rate of this functional disability varied across sub-national regions. We find that low neighborhood trust was associated with an increased likelihood of toileting disability, as was the use of assistive mobility devices. The negative effects of these social and external environment characteristics hold when we stratified the sample by rural and urban residency. Also, older adults in urban areas without access to toilets and using shared latrines had higher odds of being disabled in terms of toileting. Other factors important in explaining toileting disability among older adults included poor self-rated health, arthritis, currently working, living in the East or West region, and having functional limitations.

**Conclusions:**

Poor person-environment fit can compromise older adults’ ability to perform self-care tasks. Policymakers need to look beyond the physical environment (e.g., dedicating resources to construct toilet facilities) to adopt a more holistic, multi-faceted approach in their sanitation policies. Improving the safety of neighborhood surroundings in which shared latrines are located and the availability of accessible toilets that cater to those with mobility impairments can help improve independence in toileting among older adults.

## Introduction

Sanitation is one of the primary characteristics of a person’s physical and lived environment that is critical for one’s health and human capital development, as well as overall well-being. Yet, in many low- and middle-income countries, access to basic sanitation and open defecation remains a major public health concern [1, 2]. In particular, India has been consistently ranked as one of the countries with the lowest prevalence of access to basic sanitation [3, 4]. According to WHO/UNICEF Joint Monitoring Programme estimates for 2020, over 229 million residents in India do not have basic or shared access to toilets. Sanitation coverage in the rural areas of the country is especially lacking [5, 6]. The provision of adequate sanitation is also a challenge for India’s urban poor, who live in overcrowded slum settlements where sewerage is precarious and space for toilets is at a premium [7, 8]. About 15% of India’s population defecate in fields, forests, bushes, bodies of water, or other open spaces in 2020 [4]. Sanitation research has also documented various factors which influence the usage of toilets in India including preference for open defecation, gender norms, as well as cultural factors including caste and purity [6–8].

In relation, a large body of research links the lack of safe sanitation and open defecation to a range of health-related outcomes such as the transmission of diarrhoeal diseases such as cholera and dysentery [9, 10]; excess mortality among children under five years of age [11]; childhood stunting [12]; non-marital sexual violence among women [13–15]; as well as distress and poorer mental health [4, 16, 17]. By contrast, little is known about the impact of lack of sanitation access (e.g., no access to toilets; unsafe social environment near toilets) on older adults’ functional health particularly in the terms of toileting independence. Toileting can be defined as a sequence of related tasks which include getting to and from the toilet facility, transferring on and off the toilet or commode, being able to clean oneself afterward, as well as rearranging one’s garments upon completion [18]. Inequalities in access to safe sanitation systems, which are particularly acute in low- and middle-income countries, may disproportionately affect the older demographic due to age-related declines in physical and cognitive capacity which make older persons more vulnerable to physical and social barriers in their surroundings. Moreover, biological changes with age oftentimes bring about a need to use the toilet more frequently and also with greater urgency [19].

Yet, existing literature on toileting disability among older adults is sparse. A systematic literature review pointed out that while many empirical studies recognized toileting disability as a significant problem among older persons, these studies generally did not report the prevalence of and factors associated with toileting disability. Instead, the typical approach was to include toileting disability with other activity of daily living (ADL) items to generate an overall functional disability score [20]. Some recent studies have attempted to address this research gap. For instance, one study conducted in Ghana showed that older adults without access to toilets have much higher odds of difficulty toileting than those who have access to toilet facilities [21]. Liu et al. [22] showed that older adults living in rural villages in China were more likely to have toileting disability if toilets were located outdoors rather than indoors. Fong and Feng [23] highlighted that social environmental characteristics explained the presence of toileting disability, in addition to physical environmental barriers. That study found that older adults in China with low neighborhood trust levels and who do not feel safe at home were more likely to have a toileting disability, as were those using non-flush toilet systems or shared latrines [23]. In other words, there is growing evidence that toilet access and factors related to the toileting environment may influence older adults’ inability to perform toileting activities.

We contribute to this accumulating body of literature by examining the prevalence of toileting disability among older adults in India and its association with broad aspects of the physical and social environment. We draw on the disablement process model as the guiding framework for our analysis. Toileting is a key item in the basic ADL index and a standard clinical measure of functional disability among older persons [18, 24]. Widely referenced by researchers in studying ADL disabilities and its associated factors, the disablement process framework posits that disability occurs when there is a gap between an individual’s capabilities and environmental demands [25, 26]. Our primary interest is to evaluate such a gap exists in the context of toileting disability by evaluating the respective roles of physical and social environment characteristics on toileting independence.

Our study is thus an important extension of research on how poor person–environment fit can compromise older adults’ independence and ability to perform daily self-care activities [27–30]. Toileting disability, in particular, has garnered more global research attention than other forms of ADL disabilities in environmental gerontology because toileting is often performed in specialized built environments that require users to be safely separated from excreta. In addition, India provides an appealing setting for our investigation. India surpassed China as the world’s most populous country in 2023 [31]. India is also aging faster than previously projected. The United Nations forecasts that Indians over the age of 60 years will double by 2050, constituting almost 20% of the total population [32]. The rising number of Indian older adults has led to concerns regarding their health. Understanding the risk factors associated with toileting disability can help policymakers and health administrators identify and develop relevant intervention strategies to assist older adults in meeting their care needs, particularly in terms of addressing possible deficits in the physical and social environments in which toileting activities are conducted.

## Methods

### Data and sample

We analyzed cross-sectional data sourced from the inaugural wave of the Longitudinal Ageing Study in India (LASI), 2017-18. The LASI is a large-scale nationally representative survey of health, economic, and social well-being of the Indian population aged 45 and older, as well as their spouses. The survey was conducted from April 2017 to December 2018, covering 35 states and union territories in the country. It included a sample of 31,464 persons aged 60 and above. The LASI is internationally harmonized with the U.S. Health and Retirement Study, and contains rich information on demographics, economic status, living environment, employment, chronic health conditions, family networks, and retirement. The survey also adopted a multistage stratified cluster sampling strategy to ensure representativeness and individual-level weights are available. Computer-assisted interviews were conducted face-to-face by trained interviewers. Response rates were generally high at around 85% on average.

Ethical approval for the LASI was obtained from the Indian Council of Medical Research and all participants provided written informed consent. A detailed description of LASI is given elsewhere [33, 34]. This paper is based on secondary data analyses and all methods were performed in accordance with the relevant research guidelines and regulations. After excluding 463 subjects with missing data on toileting facility, our final analytical sample comprised 20,789 community-dwelling adults aged ≥ 65 years spread across 35 states/territories. We included proxy respondents (who account for 11% of the analytical sample) to maximize sample size and also because it is fairly common to include proxy respondents in research involving older or disabled people. Missing values (including do not know or refuse) were minimal in the dataset and generally < 5% of the sample. Thus, we imputed missing values using mean imputation methodology. Individual-level weights (available from the dataset) are applied in all analyses in order to produce nationally representative population estimates. The weighted sample was *N* = 80,608,413.

### Dependent variable

The outcome variable of interest was the presence of toileting disability. The LASI collates self-reported information on various ADL disabilities (e.g., dressing and bathing) using a standard question following the U.S. Health and Retirement Study. Specifically for the toileting ADL disability, respondents were asked: “Now, I will ask you about a few everyday activities. Please tell me if you have any difficulty with [using the toilet, including getting up and down] because of a physical, mental, emotional, or memory problem. Please exclude any difficulties you expect to last less than three months.” The answer categories provided were yes or no. Those who responded “yes” to the question were considered as having a toileting disability, as per prior studies [21–24].

### Physical and social environmental factors

We draw on the disablement process model as the guiding framework to identify risk factors associated with toileting disability in this study. In the conceptual framework, environmental variables are grouped under the ‘extra-individual factors’ domain. This domain encompasses protective or exacerbating conditions occurring in the individual’s *physical* or *social* environment, which includes the presence of *external supports* that are critical especially in late-life disability [26]. The four other key domains in the framework are namely: (i) ‘pathologies’; (ii) ‘impairments’; (iii) ‘functional limitations’; and (iv) ‘intra-individual factors’, including both socio-demographic and health/lifestyle factors. Notably, this conceptual framework is aligned with WHO’s *International Classification of Functioning*,* Health and Disability*, which emphasizes that disability is the result of the interaction of the person with the environment [35]. In theoretical work, Fong and Feng [23] demonstrated how the disablement process model can be usefully deployed to classify risk factors in the context of toileting disability. Aspects of the physical environment may include measures of access to toilet facilities and the type of facility, while aspects of the social environment including external supports may include feeling safe at home or surroundings, trust in neighbors, reliance on mobility aids, and use of vision aids.

**Fig. 1:**
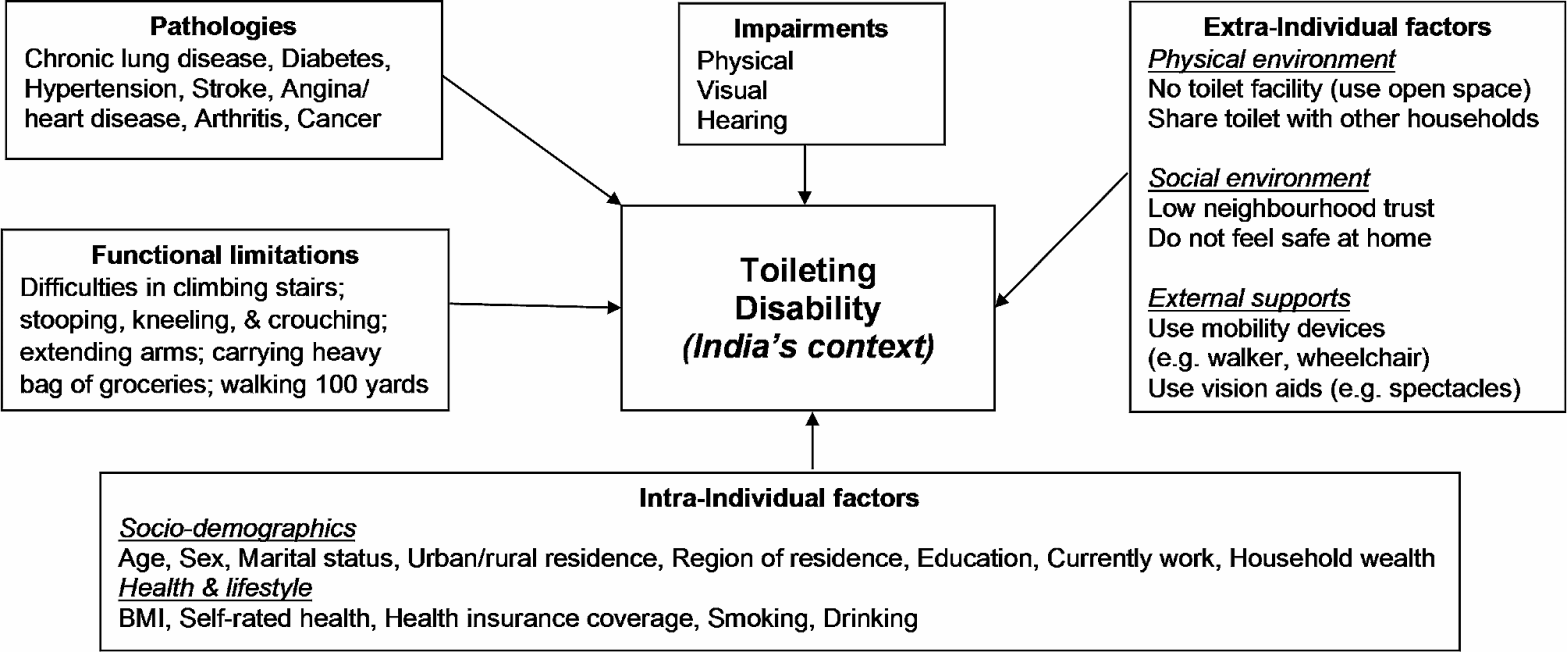
Risk factors of toileting disability based on LASI data and the disablement process conceptual framework

We adopt the same approach to identify potential risk factors of toileting disability in the LASI survey dataset (see Fig. 1). Under the ‘extra-individual factors’ domain, we identified a total of six variables related to the physical and social environment. For physical environment, we employed two separate indicator variables (1 = yes and 0 = no) for (i) lack of access to a toilet facility and (ii) sharing latrines with other households. Survey participants were asked: “What type of toilet facility does your household use?” Those who responded no facility (use open space or field) were classified as having no access to a safe sanitation system, which accounts for almost 30% of respondents in our weighted sample, confirming that this dichotomy is important to the present study.

A separate question was asked on whether respondents shared toilet facilities with other households, and those answering yes were classified as using shared latrines. The sharing of sanitation facilities is a common practice in many parts of India due to space and cost limitations. In fact, shared toilets are oftentimes the only option for many people living in the dense urban and rural areas throughout India [36–38]. For instance, Heijnen et al. [36] showed that showed that households in Orissa, India using communally-managed facilities (frequented by large numbers of households) were poorer and less educated, and the facilities were less clean and maintained as compared to households sharing a ‘localized’ sanitation facility with only their neighbors or landlord.

We account for social features of the toileting environment and usage of external supports using four distinct dummy variables. The first relates to trust in the neighborhood surroundings. Respondents were considered as having low neighborhood trust if they did not feel safe when they walked down their street/locality after dark. The second relates to safety in the immediate home environment. Specifically, LASI respondents were asked: “In general, how safe from crime and violence do you feel when you are alone at home?” Those who said not safe at all were categorized as not feeling safe at home. Use of assistive devices—external devices that are designed, made, or adapted to assist a person to perform a particular task—can influence the way that older adults operate within and interact with their physical and social environments. The LASI dataset contains a rich set of variables relating to aid or supportive devices that assist older adults in their daily life. Accordingly, we added a dummy variable for use of mobility devices (walker, walking stick, or wheelchair) and a separate dummy variable for use of vision aids (spectacles/contact lenses).

### Control variables

Control variables were also systematically identified following the defined domains in the conceptual framework. For the ‘pathologies’ domain, we used seven indicator variables measuring of whether a respondent was ever diagnosed with a certain chronic condition, including chronic lung disease, diabetes, hypertension, stroke, angina/heart disease, arthritis, and cancer. The ‘impairments’ domain comprised three dummy variables indicating physical impairment (in lower or upper body), visual impairment, and hearing impairment, respectively. Consistent with past studies [21, 23], we used separate indicators in the ‘functional limitations’ domain to account for respondents’ difficulty in climbing stairs; stooping, kneeling or crouching; and extending arms. Additionally, we included difficulty carrying a heavy bag of groceries and difficulty walking 100 yards (approximately 92 meters) since these measures that were available in the LASI dataset also pertained to functional limitations.

The ‘intra-individual factors’ domain included age (65–69, 70–74, 75–79, and ≥ 80 years); sex (1 = female); marital status; (1 = married/cohabiting); education attainment (none, primary or less, at least some secondary education); residence (1 = rural); employment status (1 = currently working); and household wealth terciles (low, middle, high). Past studies have noted a high degree of variation in socioeconomic conditions across various regions in the Indian subcontinent [39, 40]. To capture regional differentiation in socioeconomic status and its potential effects on toileting disability, we thus added region dummies (North, Northeast, East, Central, West, and South). Health and lifestyle risk factors included poor self-rated health, health insurance coverage, currently smoke, currently drink, all measured dichotomously. The five BMI categories were: underweight (BMI < 18.5), normal (18.5 ≤ BMI < 23; reference), overweight (23 ≤ BMI < 25), obese (25 ≤ BMI < 30), and severely obese (BMI ≥ 30).

### Statistical analysis

Our empirical approach began with an examination of the prevalence of toileting disability in the weighted sample. We also investigated graphically how toileting disability prevalence rate varies across states/territories in India in 2017-18. Although a correlation between state of residence and toileting disability is not a hypothesis or focus of this present paper, our descriptive result in the form of a heatmap provides a useful overview of the outcome variable (toileting disability) given that the LASI respondents are spread across 35 states/territories in India. Next, we compared the characteristics for persons with and without toileting disability, using standard statistical chi-square tests for the six environmental factors.

Hierarchical logistic regressions were implemented to analyze how the different extra-individual (environmental) factors were associated with toileting disability. Model 1 featured only the physical environment factors, model 2 only the social environment factors, and model 3 only the external supports environment factors. Model 4 contained all three types of environmental factors. The full set of control variables were used in all model specifications. We checked multicollinearity in the logistic models, and linearly combined control variables that were highly correlated (*ρ* > 0.5) following Fong and Feng [23]. The analyses were conducted using Stata version 17.0 (StataCorp LCC, College Station, United States of America), and accounted for the multistage stratified cluster sampling design and for potentially correlated data that occurs when respondents live in the same household. Sampling weights were used to ensure nationally representative population estimates, thus increasing the generalizability of the results.

## Results

### Prevalence of toileting disability

**Fig. 2:**
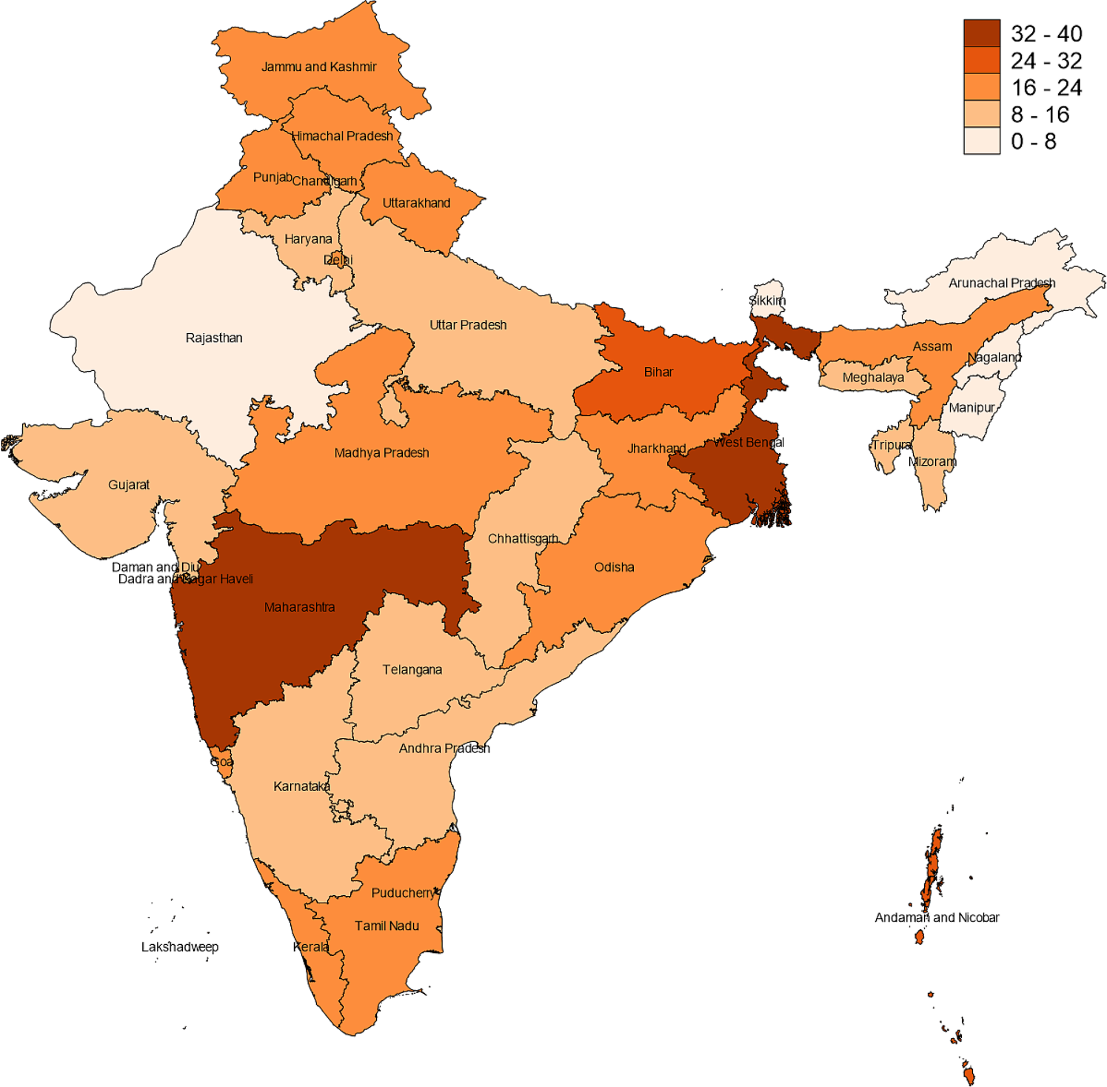
Heat map of prevalence of toileting disability by state In India

The overall prevalence of toileting disability is about 20.4% in our weighted sample. Heatmap analysis provides further insights on regional heterogeneity. Figure 2 shows the map of India’s 35 states/union territories according to levels of toileting disability prevalence. There is a distinct pattern of regional differences: we see that greater proportions of older adults in the Western and Eastern regions had difficulty in toileting compared to their counterparts elsewhere. The disability prevalence rate was highest in the West region at 30.2% and largely attributable to the state of Maharashtra. In Mumbai (capital of Maharashtra), it is estimated that 40% of the urban population of over 22 million live in slums [41]. For the East region, we note that smaller states like Goa and Daman-Diu. Bihar, West Bengal, and Jharkhand were the key contributors to the high observed disability prevalence rate of 27.1%. Notably, one in three older adults in West Bengal reported having difficulty toileting. Prevalence rates in the North, Northeast, Central, and South regions were only 11.6%, 14.8%, 15.6% and 16.1% respectively. These regional variations may accrue to a variety of underlying elements such as differences in socioeconomic conditions, settlement patterns, climate, geography, and culture. This heatmap overview provides support for our use of region dummies in subsequent regression analysis.

### Sample characteristics by disability status

Table 1 presents the demographic characteristics of the LASI participants. In the weighted sample, the mean age was 72 (SD = 6.8), 52% were females, 56% were married, and almost 60% had no formal education. 72% of respondents resided in rural areas, and about a quarter were currently working. Household wealth distribution is left-skewed with 40% in the lowest tercile, 46% in the middle tercile, and the remaining 14% in the highest wealth tercile. 26% rated their health as poor, and only 17% had some form of health insurance coverage. Hypertension, arthritis, and diabetes were the most prevalent among the chronic conditions assessed. Visual impairments and functional limitations were also fairly common among respondents. In terms of environmental factors, a substantial proportion of respondents (29%) reported having no sanitation access and 8.4% used shared latrines. 6% and 14% did not feel safe at home and in their neighborhoods, respectively. Use of vision aids was more prevalent (38%) than use of mobility devices (11%).

**Table 1:** Comparison of respondents with and without toileting disability

Characteristic	Total		Toileting disability				*P*-value
			No		Yes		
*Number of subjects (in %)*	*20*,*789*	*100%*	*16*,*542*	*79.6%*	*4*,*247*	*20.4%*	
*Environmental factors*							
No toilet facility	5,994	28.8%	4,775	28.9%	1,220	28.7%	0.924
Share latrines with other households	1,745	8.4%	1,328	8.0%	417	9.8%	0.037
Low neighborhood trust	2,799	13.5%	2,050	12.4%	750	17.7%	< 0.001
Do not feel safe at home	1,238	6.0%	978	5.9%	260	6.1%	0.816
Use mobility devices	2,301	11.1%	1,359	8.2%	942	22.2%	< 0.001
Use vision aids	7,913	38.1%	6,208	37.5%	1,705	40.2%	0.096
*Intra-individual factors*							
Mean age (SD)	72	6.8	71.7	6.3	74.7	7.8	< 0.001
Age groups: 65–69	8,499	40.9%	7,297	44.1%	1,201	28.3%	< 0.001
70–74	5,621	27.0%	4,529	27.4%	1,093	25.8%	
75–79	3,332	16.0%	2,488	15.0%	845	19.9%	
80 and above	3,335	16.0%	2,228	13.5%	1,107	26.1%	
Female	10,829	52.1%	8,372	50.6%	2,457	57.9%	< 0.001
Married/cohabiting	11,643	56.0%	9,553	57.8%	2,089	49.2%	< 0.001
Educational status: None	12,295	59.1%	9,588	58.0%	2,706	63.7%	< 0.001
Primary education or less	4,657	22.4%	3,639	22.0%	1,017	24.0%	
At least secondary education	3,838	18.5%	3,315	20.0%	523	12.3%	
Household wealth: Low	8,210	39.5%	6,498	39.3%	1,714	40.4%	0.002
Middle	9,669	46.5%	7,611	46.0%	2,065	48.6%	
High	2,908	14.0%	2,433	14.7%	468	11.0%	
Currently working	5,145	24.7%	4,630	28.0%	514	12.1%	< 0.001
Rural residence	14,908	71.7%	11,763	71.1%	3,145	74.1%	0.044
Region: North	2,603	12.5%	2,301	13.9%	302	7.1%	< 0.001
Northeast	629	3.0%	536	3.2%	93	2.2%	
East	4,891	23.5%	3,565	21.6%	1,327	31.2%	
Central	4,424	21.3%	3,735	22.6%	689	16.2%	
West	3,611	17.4%	2,519	15.2%	1,091	25.7%	
South	4,631	22.3%	3,886	23.5%	744	17.5%	
BMI: Underweight	5,885	28.3%	4,567	27.6%	1,318	31.0%	0.186
Normal	8,133	39.1%	6,600	39.9%	1,533	36.1%	
Overweight	2,615	12.6%	2,091	12.6%	524	12.4%	
Obese	8,133	39.1%	6,600	39.9%	1,533	36.1%	
Severely obese	2,615	12.6%	2,091	12.6%	524	12.4%	
Poor self-rated health	5,403	26.0%	3,681	22.3%	1,722	40.5%	< 0.001
Health insurance coverage	3,603	17.3%	3,017	18.2%	586	13.8%	< 0.001
Currently smoke	6,953	33.4%	5,611	33.9%	1,341	31.6%	0.112
Currently drink	1,465	7.0%	1,239	7.5%	227	5.3%	0.007
*Pathologies*							
Angina/ heart disease	1,195	5.7%	869	5.3%	326	7.7%	0.038
Arthritis	4,239	20.4%	2,868	17.3%	1,371	32.3%	< 0.001
Cancer	126	0.6%	81	0.5%	44	1.0%	0.005
Chronic lung disease	1,966	9.5%	1,490	9.0%	476	11.2%	0.048
Diabetes	2,896	13.9%	2,313	14.0%	584	13.8%	0.844
Hypertension	7,009	33.7%	5,358	32.4%	1,651	38.9%	< 0.001
Stroke	626	3.0%	344	2.1%	282	6.6%	< 0.001
*Impairments*							
Physical	1,259	6.1%	735	4.4%	524	12.3%	< 0.001
Visual	12,312	59.2%	9,457	57.2%	2,856	67.3%	< 0.001
Hearing	2,665	12.8%	1,947	11.8%	718	16.9%	< 0.001
*Functional limitations*							
Stooping, kneeling & crouching	12,784	61.5%	8,865	53.6%	3,919	92.3%	< 0.001
Climbing one flight of stairs	12,796	61.6%	8,991	54.4%	3,805	89.6%	< 0.001
Extending arms above shoulder	4,997	24.0%	2,766	16.7%	2,231	52.5%	< 0.001
Carrying things	9,389	45.2%	6,195	37.5%	3,193	75.2%	< 0.001
Walking long distance	8,233	39.6%	5,153	31.2%	3,080	72.5%	< 0.001

Notes: SD = standard deviation. For categorical variables, the numbers shown in parenthesis are percentages. For continuous variables, the numbers shown in parenthesis are the SDs. The *P*-values are calculated using the t-test and the chi-squared test for continuous and categorical variables, respectively. Individual-level weights applied

Notably, 20.4% of respondents had toileting disability. Comparing the characteristics of respondents with and without toileting disability, we see that older Indian adults with toileting disability tended to be older (mean age 74.7 years vs. 71.7 years), female, not married, lowly educated, not working, without health insurance coverage, and have poor self-rated health, arthritis, hypertension, or stroke (*p* < 0.001 in all cases). They were also more likely to suffer from impairments and functional limitations. Several of the environmental factors were significantly correlated with toileting disability in univariate analysis, including using shared toilet facilities (*p* = 0.037); low neighborhood trust (*p* < 0.001); use mobility devices (*p* < 0.001); and use vision aids (*p* < 0.096).

### Multivariate regressions

Table 2 shows the effects of various environmental factors (physical, social, and external supports) on toileting disability. The odds ratios (ORs) and the accompanying 95% confidence intervals are reported. Model 4 presents the full specification with all three types of environmental factors. Both the external support factors were predictive of toileting disability but had opposite effects. Use of mobility aids increased the odds of having toileting disability (OR = 1.46, *p* < 0.001), whereas use of vision aids had a protective effect and was associated with lower odds of disability (OR = 0.83, *p* < 0.05). Low neighborhood trust was also statistically significant; older Indian adults who did not feel safe in their neighborhoods were about 1.26 (*p* < 0.01) times more likely to have difficulties in toileting than their counterparts. We note that neither of the physical environmental factors (access to a facility and shared latrines) were predictive of the outcome.

**Table 2:** Odds ratios for toileting disability and different types of environmental factors

Variables	Model 1	Model 2	Model 3	Model 4
*Environmental factors*				
No toilet facility	0.95			0.95
	(0.80–1.14)			(0.79–1.14)
Share latrines with other households	1.23			1.22
	(0.96–1.57)			(0.96–1.57)
Low neighborhood trust		1.26**		1.26**
		(1.06–1.50)		(1.06–1.50)
Do not feel safe at home		1.38		1.35
		(0.73–2.61)		(0.72–2.53)
Use mobility devices			1.44***	1.44***
			(1.20–1.74)	(1.19–1.73)
Use vision aids			0.83*	0.84*
			(0.70–0.98)	(0.71–0.99)
*Control variables*				
*Age groups (ref: 65–69)*				
70–74	1.07	1.07	1.07	1.07
	(0.90–1.28)	(0.90–1.28)	(0.90–1.27)	(0.90–1.27)
75–79	1.30*	1.29*	1.26*	1.25
	(1.04–1.62)	(1.03–1.61)	(1.01–1.57)	(1.00–1.56)
80 and above	1.39**	1.38**	1.33*	1.32*
	(1.11–1.74)	(1.10–1.73)	(1.05–1.67)	(1.04–1.66)
Female	0.89	0.87	0.91	0.89
	(0.73–1.08)	(0.72–1.06)	(0.75–1.10)	(0.74–1.08)
Married/cohabiting	1.01	1.02	1.02	1.02
	(0.85–1.20)	(0.86–1.21)	(0.86–1.21)	(0.86–1.22)
*Educational status (ref: None)*				
Primary edu or less	1.08	1.09	1.11	1.10
	(0.89–1.32)	(0.90–1.32)	(0.91–1.35)	(0.90–1.35)
At least secondary edu	0.75*	0.75*	0.78*	0.78*
	(0.60–0.94)	(0.60–0.94)	(0.63–0.98)	(0.62–0.99)
*Household wealth (ref: Low)*				
Middle	1.18*	1.18*	1.19*	1.19*
	(1.01–1.37)	(1.01–1.39)	(1.01–1.40)	(1.02–1.39)
High	1.03	1.04	1.04	1.05
	(0.83–1.29)	(0.83–1.30)	(0.83–1.30)	(0.84–1.30)
Currently working	0.67***	0.67***	0.68***	0.68***
	(0.55–0.81)	(0.55–0.81)	(0.56–0.82)	(0.56–0.83)
Rural residence	1.24*	1.22*	1.18	1.20*
	(1.05–1.47)	(1.03–1.44)	(1.00–1.40)	(1.01–1.42)
*Region (ref: North)*				
Northeast	1.35*	1.39*	1.45**	1.43*
	(1.02–1.78)	(1.06–1.83)	(1.10–1.91)	(1.08–1.88)
East	2.74***	2.72***	2.85***	2.85***
	(2.20–3.40)	(2.20–3.36)	(2.31–3.52)	(2.29–3.54)
Central	1.28	1.26	1.31*	1.32*
	(0.99–1.65)	(0.98–1.63)	(1.01–1.69)	(1.02–1.71)
West	3.61***	3.56***	3.75***	3.64***
	(2.87–4.55)	(2.83–4.48)	(2.97–4.72)	(2.89–4.59)
South	1.15	1.13	1.18	1.18
	(0.93–1.43)	(0.91–1.40)	(0.95–1.46)	(0.95–1.46)
*BMI (ref: Underweight)*				
Normal	0.78**	0.78**	0.78**	0.78**
	(0.65–0.93)	(0.65–0.94)	(0.65–0.93)	(0.65–0.93)
Overweight	0.84	0.85	0.85	0.85
	(0.66–1.08)	(0.67–1.09)	(0.66–1.08)	(0.66–1.08)
Obese	0.86	0.88	0.87	0.87
	(0.69–1.08)	(0.70–1.10)	(0.70–1.10)	(0.69–1.09)
Severely obese	0.83	0.83	0.83	0.83
	(0.57–1.20)	(0.57–1.20)	(0.57–1.21)	(0.57–1.21)
Poor self-rated health	1.49***	1.49***	1.48***	1.47***
	(1.30–1.73)	(1.29–1.72)	(1.28–1.70)	(1.28–1.70)
Health insurance coverage	0.80**	0.79**	0.78**	0.79**
	(0.67–0.94)	(0.67–0.94)	(0.66–0.93)	(0.66–0.94)
Currently smoke	0.86	0.85*	0.86	0.84*
	(0.73–1.01)	(0.73–1.00)	(0.73–1.00)	(0.72–0.99)
Currently drink	1.07	1.06	1.04	1.03
	(0.79–1.46)	(0.77–1.44)	(0.76–1.42)	(0.76–1.41)
Angina/ heart disease	1.23	1.23	1.26	1.24
	(0.91–1.67)	(0.91–1.67)	(0.93–1.69)	(0.92–1.68)
Arthritis	1.45***	1.44***	1.45***	1.43***
	(1.25–1.67)	(1.24–1.67)	(1.25–1.68)	(1.23–1.66)
Cancer	1.24	1.26	1.22	1.23
	(0.69–2.20)	(0.71–2.24)	(0.68–2.19)	(0.68–2.22)
Chronic lung disease	0.85	0.84	0.85	0.84
	(0.69–1.05)	(0.68–1.04)	(0.69–1.05)	(0.68–1.04)
Diabetes	0.89	0.88	0.88	0.88
	(0.73–1.07)	(0.73–1.06)	(0.73–1.06)	(0.73–1.06)
Hypertension	1.02	1.03	1.03	1.02
	(0.88–1.19)	(0.89–1.19)	(0.89–1.19)	(0.88–1.19)
Stroke	1.32	1.34*	1.30	1.29
	(1.00–1.75)	(1.01–1.77)	(0.98–1.73)	(0.97–1.72)
Physical impairment	1.70***	1.67***	1.62***	1.61***
	(1.34–2.15)	(1.32–2.11)	(1.28–2.05)	(1.27–2.04)
Visual impairment	1.13	1.15	1.22*	1.22*
	(0.97–1.33)	(0.98–1.34)	(1.04–1.43)	(1.04–1.43)
Hearing impairment	1.08	1.06	1.06	1.05
	(0.90–1.28)	(0.89–1.27)	(0.89–1.26)	(0.88–1.25)
Climbing & stooping	2.21***	2.20***	2.20***	2.19***
	(1.85–2.63)	(1.84–2.62)	(1.85–2.62)	(1.84–2.61)
Extending arms above shoulder	1.92***	1.91***	1.93***	1.93***
	(1.66–2.23)	(1.65–2.22)	(1.67–2.24)	(1.66–2.24)
Carrying things	1.57***	1.59***	1.54***	1.55***
	(1.32–1.88)	(1.33–1.90)	(1.29–1.85)	(1.29–1.86)
Walking long distance	1.91***	1.90***	1.85***	1.87***
	(1.64–2.22)	(1.63–2.21)	(1.60–2.16)	(1.61–2.17)
*N*	*20*,*789*	*20*,*789*	*20*,*789*	*20*,*789*
*Pseudo-R2*	*0.251*	*0.251*	*0.253*	*0.254*
*AIC*	*2*,*862*	*2*,*860*	*2*,*855*	*2*,*849*

Notes: AIC = Akaike Information Criterion. ***p < 0.001, **p < 0.01, and *p < 0.05. Odds ratios from the logistic regressions are reported, together with the 95% CIs in parentheses. Individual-level weights are used in the analysis; see text

In the final model, other factors positively associated with toileting disability included being older, poor self-rated health, arthritis, physical impairment, visual impairment, and having functional limitations like difficulty climbing/stooping, extending arms, and walking long distance. Older adults who lived in the East or West region of India were also significantly more likely to have a toileting disability, controlling for other confounders. This result is consistent with the descriptive evidence shown earlier in the heatmap representation, and possibly attributable to the large slum settlements in Mumbai (capital of Maharashtra) in the West and poorer states in the East region like Bihar and Jharkhand where many people live below the poverty line. Higher education, currently working, normal BMI, and having health insurance were negatively and significantly associated with toileting disability. Comparing across columns in the Table, we note that the magnitude and statistical significance of the physical, social, and external support environmental factors when assessed singularly in columns 1–3 are largely similar to those in the final model. This stems from low correlation among the three blocks of factors; for example, the pairwise correlation between no toilet facility and low neighborhood trust is only 0.032, while that between shared latrines and use mobility aids is only − 0.004. Collinearity among the other covariates used was minimal. The only correlation exceeding 0.50 in absolute value were those between difficulty stooping and difficulty climbing (*r* = 0.63). Thus, we linearly combined them by addition resulting in a total of four (rather than five) functional limitations variables in the regressions.

### Additional analyses and stratification by place of residence

While the health impacts of social environment characteristics in our study sample are evident from above, the lack of association between physical environmental factors and toileting disability is somewhat perplexing. Moreover, the toileting ADL activity is not simply about defecation and urination, but also comprises related tasks such as getting to and from the toilet, transferring on and off the commode, and being able to clean oneself afterward [18]. As a start, we rule out the possibility of collinearity between the physical environmental factors and the other environment-type factors given their low pairwise correlations as noted above. Interestingly, a number of studies [17, 42] have documented that the lack of latrines in India is more evident in rural than urban communities with some researchers even choosing to focus solely on sanitation problems in the rural or remote parts of India. This suggests it might be valuable to stratify the sample by place of residence and re-run our main estimations for rural and urban subgroups.

Results are shown in Table 3. Importantly, the negative effects of low neighborhood trust and use of mobility devices on toileting disability hold when we stratified the sample by place of residence. Both these social environment characteristics are strong predictors of toileting disability in our sample. By contrast, the use of vision aids was positively associated with disability only in the rural subsample while the health impact of physical environmental factors is only evident in the urban subsample. Urban residents without sanitation access (OR = 1.72, *p* < 0.05) or who were using shared latrines (OR = 1.64, *p* < 0.05) had higher odds of having difficulties with toileting than their counterparts (see last column of the Table). A number of determinants were common across both rural and urban subsamples, including poor self-rated health, arthritis, currently working, living in the East or West region, and having functional limitations (including difficulty climbing/stooping, extending arms, carrying things, and walking long distance).

**Table 3:** Odds ratios for toileting disability, stratified by rural versus urban residence

Variables	Full sample	Rural subsample	Urban subsample
*Environmental factors*			
No toilet facility	0.95	0.92	1.72*
	(0.79–1.14)	(0.76–1.11)	(1.06–2.80)
Share latrines with other households	1.22	1.11	1.64*
	(0.96–1.57)	(0.84–1.49)	(1.06–2.52)
Low neighborhood trust	1.26**	1.26*	1.42*
	(1.06–1.50)	(1.03–1.53)	(1.03–1.97)
Do not feel safe at home	1.35	1.19	2.06
	(0.72–2.53)	(0.64–2.24)	(0.42–10.23)
Use mobility devices	1.44***	1.42**	1.47*
	(1.19–1.73)	(1.14–1.77)	(1.04–2.08)
Use vision aids	0.84*	0.78*	1.00
	(0.71–0.99)	(0.64–0.95)	(0.76–1.32)
*Control variables*			
*Age groups (ref: 65–69)*			
70–74	1.07	1.08	1.02
	(0.90–1.27)	(0.89–1.32)	(0.74–1.41)
75–79	1.25	1.35*	1.03
	(1.00–1.56)	(1.04–1.76)	(0.71–1.50)
80 and above	1.32*	1.36*	1.23
	(1.04–1.66)	(1.03–1.79)	(0.84–1.81)
Female	0.89	0.91	0.82
	(0.74–1.08)	(0.73–1.14)	(0.56–1.21)
Married/cohabiting	1.02	1.06	0.89
	(0.86–1.22)	(0.87–1.30)	(0.65–1.20)
*Educational status (ref: None)*			
Primary edu or less	1.10	1.08	1.19
	(0.90–1.35)	(0.84–1.39)	(0.87–1.63)
At least secondary edu	0.78*	0.90	0.68*
	(0.62–0.99)	(0.67–1.19)	(0.47–0.99)
*Household wealth (ref: Low)*			
Middle	1.19*	1.26*	1.04
	(1.02–1.39)	(1.05–1.51)	(0.77–1.40)
High	1.05	1.01	1.05
	(0.84–1.30)	(0.78–1.30)	(0.70–1.57)
Currently working	0.68***	0.73**	0.46***
	(0.56–0.83)	(0.58–0.90)	(0.30–0.72)
Rural residence	1.20*	-	-
	(1.01–1.42)		
*Region (ref: North)*			
Northeast	1.43*	1.43*	1.57
	(1.08–1.88)	(1.05–1.95)	(0.82–3.00)
East	2.85***	3.12***	2.15**
	(2.29–3.54)	(2.46–3.96)	(1.36–3.40)
Central	1.32*	1.32	1.77*
	(1.02–1.71)	(0.99–1.75)	(1.06–2.96)
West	3.64***	4.57***	2.34***
	(2.89–4.59)	(3.47–6.01)	(1.52–3.62)
South	1.18	1.35*	0.88
	(0.95–1.46)	(1.06–1.72)	(0.58–1.33)
*BMI (ref: Underweight)*			
Normal	0.78**	0.73**	1.09
	(0.65–0.93)	(0.60–0.90)	(0.76–1.56)
Overweight	0.85	0.73*	1.37
	(0.66–1.08)	(0.55–0.97)	(0.86–2.18)
Obese	0.87	0.91	0.97
	(0.69–1.09)	(0.70–1.20)	(0.64–1.48)
Severely obese	0.83	1.11	0.85
	(0.57–1.21)	(0.66–1.86)	(0.51–1.40)
Poor self-rated health	1.47***	1.48***	1.37*
	(1.28–1.70)	(1.25–1.75)	(1.05–1.78)
Health insurance coverage	0.79**	0.72**	0.97
	(0.66–0.94)	(0.59–0.88)	(0.69–1.35)
Currently smoke	0.84*	0.88	0.76
	(0.72–0.99)	(0.74–1.06)	(0.55–1.06)
Currently drink	1.03	1.11	0.60
	(0.76–1.41)	(0.78–1.57)	(0.31–1.15)
Angina/ heart disease	1.24	1.25	1.17
	(0.92–1.68)	(0.84–1.85)	(0.77–1.77)
Arthritis	1.43***	1.28**	1.88***
	(1.23–1.66)	(1.07–1.52)	(1.44–2.46)
Cancer	1.23	0.89	1.54
	(0.68–2.22)	(0.43–1.85)	(0.65–3.65)
Chronic lung disease	0.84	0.88	0.79
	(0.68–1.04)	(0.69–1.13)	(0.55–1.14)
Diabetes	0.88	0.86	0.93
	(0.73–1.06)	(0.67–1.10)	(0.70–1.24)
Hypertension	1.02	1.04	1.01
	(0.88–1.19)	(0.87–1.24)	(0.77–1.33)
Stroke	1.29	1.46*	0.97
	(0.97–1.72)	(1.03–2.06)	(0.57–1.66)
Physical impairment	1.61***	1.81***	1.18
	(1.27–2.04)	(1.41–2.33)	(0.71–1.97)
Visual impairment	1.22*	1.26*	1.11
	(1.04–1.43)	(1.05–1.51)	(0.83–1.49)
Hearing impairment	1.05	1.04	1.13
	(0.88–1.25)	(0.85–1.27)	(0.80–1.60)
Climbing & stooping	2.19***	2.05***	2.88***
	(1.84–2.61)	(1.67–2.51)	(2.23–3.72)
Extending arms above shoulder	1.93***	1.97***	1.77***
	(1.66–2.24)	(1.65–2.34)	(1.35–2.31)
Carrying things	1.55***	1.50***	1.58**
	(1.29–1.86)	(1.21–1.85)	(1.18–2.12)
Walking long distance	1.87***	2.02***	1.60**
	(1.61–2.17)	(1.70–2.40)	(1.19–2.14)
*N*	*20*,*789*	*14*,*908*	*5*,*881*
*Pseudo-R2*	*0.254*	*0.256*	*0.277*
*AIC*	*2*,*849*	*3*,*144*	*2*,*181*

Notes: AIC = Akaike Information Criterion. ***p < 0.001, **p < 0.01, and *p < 0.05. Odds ratios from the logistic regressions are reported, together with the 95% CIs in parentheses. Individual-level weights are used in the analysis; see text

## Discussion

The environments in which older adults carry out their daily activities are critical to their ability to function independently in their homes, communities, or other settings. Because toileting is a private matter and often conducted in specialized built environments or facilities, the relationships and interactions between older adults and their socio-physical environments can potentially impact their ability to use the toilet independently. Our study is the first to investigate this link in the context of India. Using a representative sample of adults aged ≥ 65 years across various states/territories in India, we found support for two hypotheses: that a poor social environment is associated with an increased likelihood of toileting disability, and that the use of assistive mobility devices is positively associated with toileting disability. Additionally, our analyses provide some support for the two hypotheses related to the positive association between toileting disability and physical environmental factors, although the evidence is largely limited to older adults residing in urban settings.

Social influences on health operate through many different processes, one of which may be the types of areas or neighborhoods in which people live. One main finding is that lower neighborhood trust increases the likelihood of toileting disability among older adults. This is broadly consistent with studies which have demonstrated the impact of neighborhood social factors on health and morbidity outcomes [43, 44]. A poor social environment can make a person feel anxious and stressed, which can lead to health conditions or diseases in the long-term. We show that in a developing country like India, where many households do not have private toilets in their homes either due to poverty or cultural norms, the social environment is an important determinant of functional disability. Many toilet facilities are built and situated outside the home in India not only because toilets are deemed unclean according to common customs and other cultural norms [13, 42], but also due to location-based characteristics or constraints, such as access to water, for flushing and self-cleaning, and soil type [6, 36]. Also, in some rural areas and urban community-managed resettlements, reliance on public toilets is substantial due to extreme poverty [7, 42]. Given such situations, older persons’ ability to perform toileting activities may be compromised if they insecure or unsafe in their community or neighborhood, especially if they require some level of human assistance with the activity.

Another main finding in this paper is that the use of mobility aids poses as an environmental barrier rather than a facilitator for older adults in the toileting activity. Our results show that respondents using a walker, walking stick, or wheelchair had significantly higher odds of having difficulties with toileting. In the full sample, the effect size (odds ratio) associated with use of mobility aids is about similar to that of low neighborhood trust. Additionally, this positive association holds for both urban or rural subsamples. Older persons may find it especially challenging to maneuver in toilets that are small or narrow. Some common bathroom hazards documented in the context of urban Indian households include inadequate door width, slippery floor, high door threshold, and the absence of grab bars [45]. Furthermore, accessible toilets designed to meet the majority of needs of independent wheelchair users and people with mobility impairments are not common in India [46].

Also pertaining to external support environmental factors, we find a weak negative association between the use of vision aids and toileting disability. Older persons, mainly rural residents, who used vision aids such as spectacles and contact lens had lower odds of toileting disability. This suggests that, unlike the use of mobility aids, the usage of vision aids is an environmental facilitator for older adults in the toileting activity. This can be rationalized since vision aids can help individuals’ see better in bathrooms with dim lighting. Our findings regarding the differential roles of external support factors on toileting disability provide a more nuanced perspective towards understanding the extent that assistive devices may alter an individual’s capabilities and serve to help narrow the competence–environment gap in toileting for older adults. While some external supports like vision aids can indeed help *narrow* the gap as what theory predicts [28, 30], other types of external supports like wheelchairs and other mobility aids may actually *widen* the person–environment gap unless architectural adaptations are made to the built environment to complement the use of such devices.

Our study confirms that deficits in the physical environment (no access to toilet facility and using shared latrines) increase the likelihood of toileting disability among older adults. What is worth noting, however, is that the evidence points to the sharper relevance of these physical environmental factors in urban settings rather than rural settings. This may be traced to a few plausible reasons. One is the existence of urban slum settlements in India. Most slum houses do not have sanitation and water facilities, either because applications for individual toilets and taps are pending approval or because the slum is on encroached land. For example, in Kolkata and Greater Mumbai, a large part of the residential area is made up of densely-populated urban slum settlements with narrow lanes characterized by overcrowding, shared intermittent water supply, shared community latrines, and sewage disposal through open drains. Even in some settlements with pit latrines, the pits often overflow exposing people to foul-smelling fecal matter [7, 8]. Another reason is that, in cities, the space for toilets and sewage systems is at a premium due to competing land use [4]. Toilets may be wholly absent in some public spaces of the city, aggravating the hardships associated with toileting for older persons who may have incontinence or need to access the toilet frequently. Lastly, in urban areas, the access to space for open defecation as an alternative to using a toilet tends to be more limited. This is unlike in rural areas where the residences are generally more spread out and often adjacent to open spaces, bushes, or bodies of water.

### Implications for policy

Over the years, the Indian government has attempted to improve access to safe sanitation over time through national sanitation programming and policy. For example, in 2003, the government introduced the Nirmal Gram Puraskar program offering cash prizes to villages, blocks, and districts that practiced proper waste management and were free from open defecation. In 2014, Indian Prime Minister Narendra Modi launched the Swachh Bharat (or Clean India) Mission in an effort to eliminate public defecation by 2019. During that five-year campaign period, a total of 110 million toilets were constructed with about 600 million people gaining access to them [47]. What is dubbed as the world’s biggest toilet-building program garnered mixed reviews. While huge progress has been made in providing toilets across hundreds of thousands of villages, not everyone has access to a toilet as yet [48, 49]. In this present study, about 29% of respondents in our weighted sample reported having no access to a toilet facility in 2017-18, and this lack of access contributes to higher odds of toileting disability among older adults in urban settlements. A caveat is that this finding is based on data collected in LASI 2017-18 when the first phase of the Swachh Bharat Mission was still ongoing. Nonetheless, our finding draws attention to the importance of a continued focus on constructing toilets that are affordable during the next phase of India’s toilet revolution, especially for the urban poor, as India’s resident population continues to age. In urban areas, efforts can focus on expanding programs for community-designed, built and managed toilet blocks that serve low-income urban dwellers, as well as ensuring access to toilets in some public spaces. Public health interventions can target to narrow the poor-rich gap in the open defecation practice among households including provisions of subsidies to the poor [1]. Where applicable, policymakers can also look into leveling certain inequalities in access such as implementing laws to prevent sewage removed from wealthier households being discharged into waterways that might pollute poorer urban residential areas.

To improve older adults’ independence in toileting, policymakers need to adopt a more holistic, multi-faceted approach in their sanitation policies beyond just dedicating resources to the construction of toilet facilities. Specifically, the association between toileting disability and low neighborhood trust we identify in this study suggests that it is important to address the underlying social environment in which latrines are located. Shared or community toilets can be made more secured by installing brighter lighting in the toilet and around the facilities, by positioning facilities closer to the households, as well as ensuring good provision for sanitation (and piped water, drainage and solid waste collection). Urban redevelopment in Indian cities need to take into account the demand for public toilets, as well as improve slum sanitation plans. In more rural areas, overcoming socio-spatial inequalities that produce social (and physical) distance should be a priority to facilitate latrine usage among older adults [6].

Although improving access to disabled-friendly toilets is one of the goals of the ambitious Swachh Bharat Mission programme, it appears that more remains to be done. Our study shows that the use of wheelchairs and other mobility aids is currently an environmental barrier for older adults in toileting. Disability is the result of the interaction of the person with the social and physical environment [35]; wheelchair accessible washrooms with adequate space, presence of grab bars, slip-resistant flooring, and adjustable wash basins are examples of architectural adaptations that can help narrow the older person–environment gap in toileting. Also, comfort-height toilets mean that older adults can exert less effort getting on and off the seat, especially those with hip, knee, joint, or back problems. Given the cost-benefit tradeoffs of sanitation programs and improvements [50], however, it will be useful to adopt a more targeted approach in the roll-out of accessible toilets in communities. Future research is required to more closely examine the outdoor, entrance, and indoor barriers that older persons using mobility aids face in toileting so that disabled-friendly toilet facilities can be further enhanced. Also, whilst we have controlled for many possible confounders in the multivariate regression framework, there may still be some omitted variables such as people’s attitudes towards open defecation. Future research can also help remedy this aspect.

## Conclusion

India is the world’s most populous country and is rapidly aging. The right to sanitation and water is recognized as fundamental to attaining all other human rights. Our study has shown that older adults in India are disproportionately affected by the lack of sanitation access, and that such deficits can contribute to their functional disability in terms of toileting. This is in addition to the various adverse health outcomes such as childhood morbidity attributable to the lack of safe sanitation that has been emphasized in the previous literature. Beyond influence from the physical environment, toileting disability among older adults is shaped by the social environment and individuals’ use of external supports. Safety and social order in the neighborhood surroundings where shared latrines are located is particularly important. Many of these environmental factors are policy-modifiable, as discussed above. Taken together with similar evidence that has emerged from other developing countries like China and Ghana, our study has important implications for policy and practice to reduce the prevalence of functional disability among older adults in the developing world.

## Data Availability

The datasets analyzed during the current study are available in the Longitudinal Aging Study in India (LASI) repository at https://www.iipsindia.ac.in/content/LASI-data.

## Abbreviations

ADL: Activity of Daily Living
LASI: Longitudinal Ageing Study in India
WHO: World Health Organization
